# Preoperative demographics and laboratory markers may be associated with early dislocation after total hip arthroplasty

**DOI:** 10.1186/s40634-023-00659-z

**Published:** 2023-10-06

**Authors:** Brandon E. Lung, Megan R. Donnelly, Kylie Callan, Maddison McLellan, Taha Taka, Russell N. Stitzlein, William C. McMaster, David H. So, Steven Yang

**Affiliations:** Irvine School of Medicine, Department of Orthopaedic Surgery, University of California, 101 The City Drive South, Pavilion III, Building 29A, Orange, CA 92868 USA

**Keywords:** Total hip arthroplasty, Prosthetic dislocation, Medical optimization, Frailty, Postoperative complication

## Abstract

**Purpose:**

The purpose of this study was to identify modifiable medical comorbidities, laboratory markers and flaws in perioperative management that increase the risk of acute dislocation in total hip arthroplasty (THA) patients.

**Methods:**

All THA with primary indications of osteoarthritis from 2007 to 2020 were queried from the National Surgical Quality Improvement Program (NSQIP) database. Demographic data, preoperative laboratory values, recorded past medical history, operative details as well as outcome and complication information were collected. The study population was divided into two cohorts: non-dislocation and dislocation patients. Statistics were performed to compare the characteristics of both cohorts and to identify risk factors for prosthetic dislocation (*α* < 0.05).

**Results:**

275,107 patients underwent primary THA in 2007 to 2020, of which 1,258 (0.5%) patients experienced a prosthetic hip dislocation. Demographics between non-dislocation and dislocation cohorts varied significantly in that dislocation patients were more likely to be female, older, with lower body mass index and a more extensive past medical history (all *p* < 0.05). Moreover, hypoalbuminemia and moderate/severe anemia were associated with increased risk of dislocation in a multivariate model (all *p* < 0.05). Finally, use of general anesthesia, longer operative time, and longer length of hospital stay correlated with greater risk of prosthetic dislocation (all *p* < 0.05).

**Conclusions:**

Elderly female patients and patients with certain abnormal preoperative laboratory values are at risk for sustaining acute dislocations after index THA. Careful interdisciplinary planning and medical optimization should be considered in high-risk patients as dislocations significantly increase the risk of sepsis, cerebral vascular accident, and blood transfusions on readmission.

## Background

Total hip arthroplasty (THA) is a common surgical procedure that has been considered one of the most cost-effective treatments for hip osteoarthritis by decreasing pain, improving mobility and function as measured by multiple patient-reported outcome scores. THA has provided long term improvement in patient quality of life with more than 95% survival rate of hip arthroplasties after 10 years [1]. While rare, one of the most common complications of THA is prosthetic hip dislocation which is the primary cause of readmission within 90 days after the surgery [2, 3].

Prosthetic hip dislocation is typically an early post-operative period complication with first-year dislocation rates ranging from 0.5 to 1.5% with some variation reported with different surgical approaches [4–6]. Several mechanisms have been proposed and are believed to contribute to the dislocating event which include the following: 1) mispositioning or loosening of the prosthesis, 2) contact between the neck of the prosthesis with the articular component, 3) contact between the bony femur and bony pelvis, and lastly, 4) hyperlaxity of the surrounding musculature or tissue [4–6].

Due to the unfavorable effect that hip dislocations have on patient satisfaction and surgical outcome, a strong effort has been dedicated to identifying risk factors which predispose patients to dislocations. Of note, this effort has resulted in the identification of several patient-associated factors including previous hip surgery, patient non-compliance, neuromuscular and cognitive disorders, smoking/chronic obstructive pulmonary disease (COPD), ASA class of 3 to 5, fracture, elevated creatinine (Cr), age ≥ 80 years, chronic steroid use, longer operative duration, and general anesthesia [7]. Additionally, several patient-associated risk factors have been shown to affect overall patient outcomes in THA, including frailty, age, malnourishment, and medical comorbidities (e.g., anemia, chronic kidney disease [CKD]) but a direct relationship with post-operative hip dislocation rate has either not been investigated or has not been consistently demonstrated across studies [8, 9]. For example, a previous study has concluded an increased risk of mortality and perioperative complications in primary and revision hip arthroplasty patients [10]. Similarly, a previous study by Eminovic et al. determined an increased hospital stay and post-operative complications in malnourished patients undergoing elective THA [11]. In both studies, no specific relationship was characterized with post-operative hip dislocations.

Understanding the several risk factors that increases a patient’s propensity for post-operative hip dislocation is crucial for patient candidacy, pre-operative planning, intra operative implementation, and post-operative precautions. For this reason, our investigation aims to utilize the National Surgical Quality Improvement Program (NSQIP) which provides an extensive database with a large sample size to further identify other patient-related risk factors associated with prosthetic hip dislocation such as dehydration, malnourishment, frailty index, severity of anemia, and other patient-associated risk factors.

## Methods

The NSQIP database was queried for all primary THA performed from 2007 to 2020. As the database was de-identified, this study was exempt from approval by the Institutional Review Board and thus informed consent was not obtained. The NSQIP database is a well-known and well-utilized resource. It has been used for many research studies related to general orthopedics, including hip arthroplasty [12–15]. The database contains patient information from greater than 600 hospitals across the United States (US). Data is obtained and uploaded by certified health care professionals using outpatient visits, direct interviews and reviews of postoperative medical record notes [16]. The inter-reliability disagreement for this data has been estimated to be < 2% [17]. Additionally, the database is audited at regular intervals helping to ensure its accuracy [18].

Current Procedural Terminology (CPT) code 27,130 was used to identify patients who underwent THA from 2007 to 2020. Patients aged ≥ 18 years with documented past medical history, preoperative laboratory values as well as reported outcomes and complications were included in this study. Patients younger than 18 years of age and those without outcome and complication data were excluded. 275,107 subjects met the inclusion criteria for the study and thus were included in statistical analysis. Demographic data, preoperative laboratory values, recorded past medical history, THA operative details as well as outcome and complication information were collected. As this study sought to evaluate the risk factors for prosthetic hip dislocation, the study population was divided into two cohorts: non-dislocation and dislocation patients. This was done using the International Classification of Diseases, Ninth (996.4, 996.5, 996.6, 996.7) and Tenth Revisions (M24 and T84), Clinical Modification codes for prosthetic hip dislocation.

Laboratory values included sodium (normal 135 to 147 mmol/l), eGFR (normal ≥ 90 ml/min/1.73m^2^, mild/moderate 30 to 89 ml/min/1.73m^2^, severe < 30 ml/min/1.73m^2^), WBC (low/normal 0 to 11 × 10^9^/l, high 12 +  × 10^9^/l), and platelets (low 0 to 139 × 10.^9^/l). Levels of anemia were stratified by hematocrit levels with nonanemia (hematocrit > 36% for women, > 39% for men), mild anemia (hematocrit 33%-36% for women, 33%-39% for men), and moderate to severe anemia (hematocrit < 33% for both women and men). The BUN/Cr ratio has been validated as a sensitive marker for predicting dehydration, and severity of dehydration was defined as Bun/Cr < 20 (non-dehydrated), 20 ≤ Bun/C ≤ 25 (moderately-dehydrated), 25 < Bun/Cr (severely-dehydrated). Hypoalbuminemia has been validated in the literature as a marker for malnutrition and is defined as levels < 35 g/l [13]. The 5-factor modified frailty index (mFI-5) has been previously validated in joint arthroplasty to correlate with increased morbidity and mortality [19]. The frailty score is a summation of presence of five comorbid variables, including congestive heart failure, diabetes mellitus, chronic obstructive pulmonary disease or current pneumonia, hypertension requiring medication, and non-independent functional status [19].

Bivariate and multivariate analyses of risk factors for prosthetic dislocation were performed. Comparative analyses were conducted using Chi-squared or Fischer’s exact test for categorical variables and student’s t-test for continuous variables. To evaluate the correlation between laboratory values and prosthetic dislocation, bivariate logistic regression analyses were performed. Multivariate analyses were then completed using stepwise logistic regression as well as clinical judgment to identify the best fit model for all demographic, preoperative laboratory values, past medical history and operative detail variables. Furthermore, additional multivariate stepwise logistic regression analyses were performed for medical complications to identify possible confounding variables for prosthetic dislocation. Statistical significance was set as *p* < 0.05. Statistics were performed on IBM SPSS Statistics, Version 26 (IBM Corp., Armonk, NY).

## Results

### Subject characteristics

This study included 275,107 patients who underwent primary THA between the years 2007 and 2020. Of these subjects, 1,258 (0.5%) experienced a postoperative prosthetic hip dislocation. A greater proportion of the dislocation patients were female (all patients: 55.0% female, dislocation patients: 59.7% female, *p* = 0.004) and Hispanic (all patients: 3.8% Hispanic, dislocation patients: 5.7% Hispanic, *p* = 0.001). The distribution of races and body mass index (BMI) categories varied significantly between non-dislocation and dislocation groups (all *p* < 0.05). On average, dislocation patients were older (all patients: 65.4 ± 11.4 years, dislocation patients: 68.1 ± 13.3 years, *p* < 0.001) with lower BMI (all patients: 30.2 ± 6.3 BMI, dislocation patients: 28.9 ± 6.8 BMI, *p* < 0.001) (Table 1).

**Table 1:** Preoperative descriptive statistics

Variable	All Patients	Dislocation (*n* = 1,258)	*p*
n, (%)	275,107 (100.0)	1,258 (0.5)	–
Demographics			
Sex, n (%)			**0.004**
Male	123,829 (45.0)	507 (40.3)	
Female	151,194 (55.0)	750 (59.7)	
Non-Binary	4 (0.0)	0 (0.0)	
Race, n (%)			** < 0.001**
White	200,305 (72.9)	978 (77.9)	
Black/African American	21,242 (7.7)	102 (8.1)	
Asian	4,099 (1.5)	31 (2.5)	
Native Hawaiian/Pacific Islander	651 (0.2)	2 (0.2)	
American Indian/Alaska Native	1,090 (0.4)	11 (0.9)	
Unknown/Not Reported	47,454 (17.3)	131 (10.4)	
Other Race	32 (0.0)	0 (0.0)	
Race Combinations with Low Frequency	1 (0.0)	0 (0.0)	
Ethnicity, n (%)			**0.001**
Non-Hispanic	216,932 (96.2)	1,058 (94.3)	
Hispanic	8,632 (3.8)	64 (5.7)	
Age, mean ± SD	65.4 ± 11.4	68.1 ± 13.3	** < 0.001**
Height, mean ± SD	66.2 ± 4.2	65.5 ± 4.3	** < 0.001**
Weight, mean ± SD	188.7 ± 46.0	177.6 ± 48.4	** < 0.001**
BMI, mean ± SD	30.2 ± 6.3	28.9 ± 6.8	** < 0.001**
BMI Categories			** < 0.001**
BMI < 18.5	2,429 (0.9)	42 (3.4)	
18.5–24.9	52,023 (19.1)	337 (27.1)	
25–29.9	91,011 (33.3)	372 (30.0)	
30–39.9	108,163 (39.6)	421 (33.9)	
> 40	19,458 (7.1)	70 (5.6)	
Preoperative Laboratory Values			
eGFR Levels, n (%)			** < 0.001**
Normal	93,162 (36.2)	425 (36.3)	
Mild/Moderate	162,008 (63.0)	718 (61.4)	
Severe	2,121 (0.8)	27 (2.3)	
Sodium Levels, n (%)			** < 0.001**
Low	12,214 (4.8)	109 (9.2)	
Normal	242,366 (95.1)	1,067 (90.5)	
High	352 (0.1)	3 (0.3)	
BUN, mean ± SD	17.9 ± 7.3	18.5 ± 8.6	0.011
Serum Creatinine, mean ± SD	0.9 ± 0.4	1.0 ± 0.6	0.022
BUN/Cr Level, n (%)			0.039
< 20	125,362 (52.7)	581 (51.1)	
20–25	62,776 (26.4)	283 (24.9)	
25 +	49,952 (21.0)	273 (24.0)	
BUN/Cr > 10, n (%)	112,728 (47.3)	556 (48.9)	0.293
Hypoalbuminemia, n (%)	7,580 (5.3)	148 (21.0)	** < 0.001**
Total Bilirubin, mean ± SD	0.6 ± 0.4	0.6 ± 0.5	0.420
SGOT, mean ± SD	24.0 ± 15.8	24.1 ± 14.4	0.962
Alkaline Phosphatase Levels, n (%)			** < 0.001**
< 44	3,670 (2.8)	18 (2.8)	
44–147	123,068 (94.6)	576 (89.3)	
148 +	3,377 (2.6)	51 (7.9)	
WBC Level, n (%)			** < 0.001**
Low/Normal	253,832 (97.3)	1,143 (95.4)	
High	6,929 (2.7)	55 (4.6)	
Anemia Severity, n (%)			** < 0.001**
Non-Anemia	225,007 (85.8)	758 (62.9)	
Mild Anemia	28,657 (10.9)	265 (22.0)	
Moderate/Severe Anemia	8,595 (3.3)	182 (15.1)	
Low Platelet, n (%)	7,855 (3.0)	46 (3.8)	0.095
PTT, mean ± SD	29.4 ± 5.0	29.7 ± 6.3	0.247
INR, mean ± SD	1.0 ± 0.3	1.1 ± 0.3	** < 0.001**
PT, mean ± SD	11.9 ± 2.5	12.1 ± 1.7	
Past Medical History			
Diabetes, n (%)			** < 0.001**
Not Diabetic	241,555 (87.8)	1,058 (84.1)	
Insulin Dependent	8,063 (2.9)	70 (5.6)	
Non-Insulin Dependent	25,116 (9.1)	126 (10.0)	
Oral Medication	373 (0.1)	4 (0.3)	
Smoking Status, n (%)			**0.006**
Non-Smoker	240,774 (87.5)	1,069 (85.0)	
Smoker	34,333 (12.5)	189 (15.0)	
Dyspnea, n (%)			0.011
None	262,265 (95.3)	1,178 (93.6)	
At Rest	546 (0.2)	2 (0.2)	
Moderate Exertion	12,296 (4.5)	78 (6.2)	
History of Severe COPD, n (%)	10,897 (4.0)	99 (7.9)	** < 0.001**
Ascites, n (%)	73 (0.0)	0 (0.0)	1.000
CHF (in 30 days before surgery), n (%)	1,108 (0.4)	11 (0.9)	0.021
Hypertension Requiring Medication, n (%)	152,514 (55.4)	761 (60.5)	** < 0.001**
Renal Failure, n (%)	186 (0.1)	2 (0.2)	0.209
Currently on Dialysis (Preop), n (%)	697 (0.3)	14 (1.1)	** < 0.001**
Disseminated Cancer, n (%)	1,170 (0.4)	9 (0.7)	0.122
Steroid Use for Chronic Condition, n (%)	10,225 (3.7)	54 (4.3)	0.263
> 10% Loss Body Weight in Last 6 Months, n (%)	683 (0.2)	11 (0.9)	** < 0.001**
Bleeding Disorders, n (%)	6,221 (2.3)	52 (4.1)	** < 0.001**
Transfusion > 4 Units PRBCs in 72 Hours Before Surgery, n (%)	481 (0.2)	17 (1.4)	** < 0.001**
Preoperative Systemic Sepsis, n (%)			** < 0.001**
None	273,344 (99.4)	1,230 (98.0)	
SIRS	1,526 (0.6)	7 (0.6)	
Sepsis	95 (0.0)	15 (1.2)	
Septic Shock	10 (0.0)	3 (0.2)	
Frailty, n (%)			** < 0.001**
< 2	236,317 978 (77.7)	978 (86.3)	
≥ 2	37,532 (22.3)	280 (13.7)	
Operative Details			
Location, n (%)			** < 0.001**
Outpatient	18,000 (6.5)	34 (2.7)	
Inpatient	257,107 (93.5)	1224 (97.3)	
Anesthesia, n (%)			** < 0.001**
Epidural	1,656 (0.6)	12 (1.0)	
General	130,519 (47.4)	870 (69.2)	
Local	65 (0.0)	0 (0.0)	
None	40 (0.0)	0 (0.0)	
Other	147 (0.1)	2 (0.2)	
Regional	5,186 (1.9)	15 (1.2)	
Spinal	99,084 (36.0)	268 (21.3)	
MAC/IV Sedation	38,360 (13.9)	91 (7.2)	
Unknown	35 (0.0)	0 (0.0)	
ASA Class, n (%)			
0 = None Assigned	24 (0.0)	0 (0.0)	
1 = No Disturb	9,859 (3.6)	23 (1.8)	
2 = Mild Disturb	142,500 (51.8)	438 (34.8)	
3 = Severe Disturb	116,506 (42.4)	716 (56.9)	
4 = Life Threat	5,925 (2.2)	81 (6.4)	
5 = Moribund	21 (0.0)	0 (0.0)	
Operative Time, mean ± SD	91.9 ± 39.1	137.4 ± 71.1	** < 0.001**

With regards to preoperative laboratory values, the non-dislocation and dislocation groups had many differences. A greater percentage of dislocation patients had severe estimated glomerular filtration rate (eGFR) levels (all patients: 0.8% severe eGFR, dislocation patients: 2.3% severe eGFR, *p* < 0.001) as well as low sodium levels (all patients: 4.8% hyponatremic, dislocation patients: 9.2% hyponatremic, *p* < 0.001). Moreover, dislocation patients had higher average blood urea nitrogen (BUN) (all patients: 17.9 ± 7.3 BUN, dislocation patients: 18.5 ± 8.6 BUN, *p* = 0.011), serum Cr (all patients: 0.9 ± 0.4 Cr, dislocation patients: 1.0 ± 0.6 Cr, *p* = 0.002) as well as international normalized ratio (INR) (all patients: 1.0 ± 0.3 INR, dislocation patients: 1.1 ± 0.3 INR, *p* < 0.001). A larger proportion of dislocation patients had *a* < 20 BUN/Cr level (all patients: 52.7%, dislocation patients: 51.1%) as well as a 25 + BUN/Cr level (all patients: 21.0%, dislocation patients: 24.0%) (*p* = 0.039). Similarly, subjects who experienced a prosthetic dislocation after primary THA had a greater distribution of patients who were hypoalbuminemic (all patients: 5.3% hypoalbuminemic, dislocation patients: 21.0% hypoalbuminemic, *p* < 0.001), with alkaline phosphatase levels 148 + (all patients: 2.6%, dislocation patients: 7.9%, *p* < 0.001), high white blood cell (WBC) count (all patients: 2.7% with leukocytosis, dislocation patients: 4.6% with leukocytosis, *p* < 0.001) and mild to severe anemia (all patients: 14.2% anemic, dislocation patients: 37.1% anemic, *p* < 0.001) (Table 1).

For subject past medical history, dislocation patients were more likely to be insulin dependent (all patients: 2.9% insulin dependent, dislocation patients: 5.6% insulin dependent) as well as non-insulin dependent diabetics (all patients: 9.1% non-insulin dependent, dislocation patients: 10.0% non-insulin dependent) (*p* < 0.001). Dislocation subjects had larger proportions of smokers (all patients: 12.5% smokers, dislocation patients: 15.0% smokers, *p* = 0.006), patients reporting dyspnea on moderate exertion (all patients: 4.5% with dyspnea, dislocation patients: 6.2% with dyspnea, *p* = 0.011) as well as patients with severe COPD, congestive heart failure (CHF), hypertension requiring medication and, finally, patients currently on dialysis (all *p* < 0.05). Additionally, a greater percentage of dislocation patients had lost > 10% of their body weight in the last 6 months (all patients: 0.2% with weight loss, dislocation patients: 0.9% with weight loss, *p* < 0.001), had a history of a bleeding disorder (all patients: 2.3% with a bleeding disorder, dislocation patients: 4.1% with a bleeding disorder, *p* < 0.001) and who required a transfusion of > 4 units of packed red blood cells (pRBCs) in the 72 h before surgery (all patients: 0.2% transfused, dislocation patients: 1.4% transfused). A significantly larger proportion of dislocation patients experienced some form of preoperative systemic sepsis (i.e., systemic inflammatory response syndrome [SIRS], sepsis, septic shock) prior to primary THA (all patients: 0.6% septic, 2.0% septic, *p* < 0.001). Using the mFI-5 clinical frailty scale, which is a validated measure used to quantify the degree of disability from geriatric frailty, more dislocation patients had a score of ≥ 2 (all patients: 13.7% frail, dislocation patients: 22.3% frail, *p* < 0.001), which indicates greater disability (Table 1).

With regards to operative details, a greater proportion of dislocation patients had inpatient surgery (all patients: 93.5% inpatient, dislocation patients: 97.3% inpatient, *p* < 0.001) under general anesthesia (all patients: 47.4% general anesthesia, dislocation patients: 69.2% general anesthesia, *p* < 0.001). More dislocation patients had an ASA class of > 2 (all patients: 44.6%, dislocation patients: 63.3%, *p* < 0.001) and had a longer average operative time (all patients: 91.9 ± 39.1 min, dislocation patients: 137.4 ± 71.1 min, *p* < 0.001) (Table 1).

### Postoperative outcomes and complications

On average, dislocation patients had a significantly longer length of stay (LOS) in the hospital (all patients: 2.4 ± 3.1 days, dislocation patients: 4.3 ± 4.5 days, *p* < 0.001). Much fewer dislocation patients were able to be discharged to home (all patients: 83.3% discharged home, dislocation patients: 60.5% discharged home, *p* < 0.001). With regards to complications, a greater proportion of dislocation patients experienced deep incision wound surgical site infections (SSI) (all patients: 0.2% SSI, dislocation patients: 0.6% SSI, *p* = 0.019) as well as organ/space SSI (all patients: 0.3% SSI, dislocation patients: 1.4% SSI, *p* < 0.001). Dislocation patients also had higher rates of requiring a ventilator for > 48 h postoperatively (all patients: 0.1%, dislocation patients: 0.3%, *p* = 0.008) in addition to acute renal failure, urinary tract infection, stroke/cerebral vascular accident (CVA), postoperative coma for > 24 h, transfusion, deep vein thrombosis (DVT)/thrombophlebitis and sepsis and septic shock complications (all *p* < 0.05). Dislocation patients were almost 3 times as likely to be readmitted and require reoperation (all patients: 3.8%, dislocation patients: 10.5%, *p* < 0.001) (Table 2).

**Table 2:** Additional postoperative complications

Outcomes	All Patients (*n* = 275,107)	Dislocation (*n* = 1,258)	*p*
Length of Stay, mean ± SD	2.4 ± 3.1	4.3 ± 4.5	** < 0.001**
Discharge Destination, n (%)			** < 0.001**
Skilled Care, Not Home	26,796 (10.0)	270 (22.7)	
Unskilled Facility Not Home	368 (0.1)	3 (0.3)	
Facility Which Was Home	941 (0.4)	18 (1.5)	
Home	222,185 (83.3)	719 (60.5)	
Separate Acute Care	778 (0.3)	8 (0.7)	
Rehab	15,315 (5.7)	164 (13.8)	
Expired	199 (0.1)	3 (0.3)	
Against Medical Advice	58 (0.0)	0 (0.0)	
Hospice	47 (0.0)	2 (0.2)	
Unknown	61 (0.0)	1 (0.1)	
Multi-level Senior Community	32 (0.0)	0 (0.0)	
Superficial Wound Infections, n (%)	1,931 (0.7)	14 (1.1)	0.088
Deep Incisional SSI Complications, n (%)	583 (0.2)	7 (0.6)	0.019
Organ/Space SSI Complications, n (%)	846 (0.3)	18 (1.4)	** < 0.001**
Unplanned Intubation Complications, n (%)	430 (0.2)	3 (0.2)	0.453
Pulmonary Embolism Complications, n (%)	719 (0.3)	5 (0.4)	0.272
On Ventilator > 48 Hours Complications, n (%)	172 (0.1)	4 (0.3)	**0.008**
Progressive Renal Insufficiency Complications, n (%)	256 (0.1)	3 (0.2)	0.114
Acute Renal Failure Complications, n (%)	141 (0.1)	5 (0.4)	**0.001**
Urinary Tract Infection Complications, n (%)	2,522 (0.9)	24 (1.9)	**0.001**
Stroke/CVA Complications, n (%)	278 (0.1)	5 (0.4)	0.010
Coma > 24 Hours Complications, n (%)	2 (0.0)	1 (0.3)	0.011
Peripheral Nerve Injury Complications, n (%)	24 (0.0)	0 (0.0)	1.000
Cardiac Arrest Requiring CPR Complications, n (%)	243 (0.1)	2 (0.2)	0.305
Myocardial Infarction Complications, n (%)	663 (0.2)	5 (0.4)	0.237
Bleeding Transfusions Complications, n (%)	16,828 (6.1)	313 (24.9)	** < 0.001**
DVT/Thrombophlebitis Complications, n (%)	1,019 (0.4)	12 (1.0)	**0.003**
Sepsis Complications, n (%)	706 (0.3)	14 (1.1)	** < 0.001**
Septic Shock Complications, n (%)	173 (0.1)	3 (0.2)	0.046
Reop/Read, n (%)	10,536 (3.8)	132 (10.5)	** < 0.001**
C. Diff Occurrences, n (%)	240 (0.1)	3 (0.3)	0.085

### Multivariate and bivariate regression results

In a bivariate logistic model, severe eGFR (OR: 2.814, 95% CI: 1.902–4.161), low sodium (OR: 2.036, 95% CI: 1.671–2.482), higher preoperative BUN (OR: 1.010, 95% CI: 1.004–1.017), higher preoperative serum Cr (OR: 1.139, 95% CI: 1.049–1.235), a BUN/Cr ratio of 25 + (OR: 1.180, 95% CI: 1.022–1.363), hypoalbuminemia (OR: 4.847, 95% CI: 4.038–5.819), an alkaline phosphatase > 148 (OR: 3.261, 95% CI: 2.444–4.351), higher WBC count (OR: 1.769, 95% CI: 1.348–2.321), mild (OR: 2.761, 95% CI: 2.400–3.178) and moderate/severe anemia (OR: 6.400, 95% CI: 5.436–7.535) and higher preoperative INR (OR: 1.271, 95% CI: 1.118–1.445) were all individually associated with a greater risk of prosthetic hip dislocation following primary THA (all *p* < 0.05) (Table 3).

**Table 3:** Preoperative laboratory values and prosthetic dislocation

	OR	95% CI	p
Laboratory Values			
Preoperative eGFR (ref = normal)			
Mild/Moderate eGFR	0.971	(0.861, 1.095)	0.636
Severe eGFR	2.814	(1.902, 4.161)	** < 0.001**
Preoperative Sodium (ref = normal)			
Low Sodium	2.036	(1.671, 2.482)	** < 0.001**
High Sodium	1.944	(0.623, 6.066)	0.252
Preoperative BUN	1.010	(1.004, 1.017)	**0.002**
Preoperative Serum Creatinine	1.139	(1.049, 1.235)	**0.002**
Preoperative BUN/Cr (ref = less than 20)			
BUN/Cr 20–25	0.973	(0.843, 1.121)	0.702
BUN/Cr 25 +	1.180	(1.022, 1.363)	0.024
Preoperative Hypoalbumenia (ref = normal)	4.847	(4.038, 5.819)	** < 0.001**
Preoperative Total Bilirubin	0.904	(0.725, 1.126)	0.367
Preoperative SGOT	1.000	(0.995, 1.005)	0.962
Preoperative Alkaline Phosphatase (ref = 44–147)			
Less than 44	1.048	(0.655, 1.678)	0.845
Greater than 148	3.261	(2.444, 4.351)	** < 0.001**
Preoperative WBC Level	1.769	(1.348, 2.321)	** < 0.001**
Preoperative Anemia (ref = normal)			
Mild Anemia	2.761	(2.400, 3.178)	** < 0.001**
Moderate/Severe Anemia	6.400	(5.436, 7.535)	** < 0.001**
Preoperative Low Platelet Level (ref = normal)	1.286	(0.957, 1.728)	0.096
Preoperative PTT	1.011	(0.996, 1.027)	0.146
Preoperative INR	1.271	(1.118, 1.445)	** < 0.001**
Preoperative PT	1.039	(0.959, 1.125)	0.355

In a multivariate logistic model for BMI levels and prosthetic dislocation risk, the underweight patients were more at risk for dislocation (OR: 2.699, 95% CI: 1.953–3.729, *p* < 0.001) whereas the overweight (OR: 0.629, 95% CI: 0.543–0.730, *p* < 0.001), obese class I & 2 (OR:0.599, 95% CI: 0.519–0.692, *p* < 0.001) and obese class III (OR: 0.554, 95% CI: 0.428–0.717, *p* < 0.001) were less at risk compared to healthy BMI patients [14] (Table 4).

**Table 4:** BMI and dislocation

	OR	95% CI	*p*
BMI (ref = 18.5–24.9)			
< 18.5	2.699	(1.953, 3.729)	** < 0.001**
25–29.9	0.629	(0.543, 0.730)	** < 0.001**
30–39.9	0.599	(0.519, 0.692)	** < 0.001**
> 40	0.554	(0.428, 0.717)	** < 0.001**

Furthermore, in a multivariate logistic model examining the effects of demographic data, preoperative laboratory values, recorded past medical history, THA operative details and select outcomes on risk for prosthetic hip dislocation, multiple variables were associated with greater risk. For instance, hypoalbuminemia (OR: 2.174, 95% CI: 1.650–2.864), moderate/severe anemia (OR: 2.098, 95% CI: 1.554–2.832), general anesthesia (OR: 2.092, 95% CI: 1.640–2.668), longer operative time (OR: 1.007, 95% CI: 1.006–1.008) and longer length of hospital stay (OR: 1.012, 95% CI: 1.003–1.021) all correlated with increased risk for prosthetic hip dislocation (all p < 0.05). Additionally, American Indian race was associated with greater risk for dislocation (OR: 2.459, 95% CI:0.998,6.055), *p* = 0.050). Contrarily, discharge to home was associated with decreased risk of dislocation (OR: 0.582, 95% CI: 0.469–0.722, *p* < 0.001) (Table 5).

**Table 5:** Risk factors for dislocation

	OR	95% CI	p
Female	0.900	(0.732, 1.106)	0.316
American Indian	2.459	(0.998, 6.055)	**0.050**
BMI < 18.5	1.697	(0.931, 3.093)	0.084
Preoperative Mild/Moderate eGFR	0.989	(0.792, 1.236)	0.925
Preoperative Severe eGFR	1.171	(0.467, 2.935)	0.737
Preoperative Low Sodium	1.294	(0.921, 1.818)	0.138
Preoperative High Sodium	1.043	(0.143, 7.614)	0.967
Preoperative Creatinine	0.959	(0.775, 1.186)	0.700
Preoperative BUN/Cr 20–25	0.892	(0.696, 1.144)	0.369
Preoperative BUN/Cr 25 +	1.121	(0.873, 1.438)	0.371
Preoperative Hypoalbuminemia	2.174	(1.650, 2.864)	** < 0.001**
Preoperative Alkaline Phosphatase 148 +	1.305	(0.874, 1.950)	0.193
Preoperative High WBC Level	1.009	(0.650, 1.567)	0.967
Preoperative Moderate/Severe Anemia	2.098	(1.554, 2.832)	** < 0.001**
Preoperative Low Platelet Level	0.805	(0.517, 1.254)	0.338
Preoperative INR	1.112	(0.815, 1.516)	0.503
Insulin Dependent Diabetes	1.378	(0.920, 2.064)	0.120
Smoking Status	1.071	(0.812, 1.413)	0.625
CHF in 30 Days Before Surgery	0.737	(0.293, 1.852)	0.516
Bleeding Disorders	1.172	(0.776, 1.768)	0.450
Transfusion > 4 Units PRBCs in 72 Hours Before Surgery	1.196	(0.560, 2.555)	0.644
Frailty ≥ 2	1.268	(0.981, 1.640)	0.069
Outpatient vs Inpatient	1.915	(0.941, 3.896)	0.073
Epidural Anesthesia	1.363	(.078, 8.144)	0.305
General Anesthesia	2.092	(1.640, 2.668)	** < 0.001**
Severe Disturb ASA	1.225	(0.996, 1.506)	0.055
Operative Time	1.007	(1.006, 1.008)	** < 0.001**
Length of Total Hospital Stay	1.012	(1.003, 1.021)	**0.011**
Discharge, Home	0.582	(0.469, 0.722)	** < 0.001**

Lastly, in a multivariate model for risk of postoperative complications associated with prosthetic dislocation, stroke/CVA (OR: 2.894, 95% CI: 1.181–7.093), bleeding requiring transfusion (OR: 5.092, 95% CI: 4.475–5.794) and sepsis (OR: 3.232, 95% CI: 1.887–5.536) were significant (all *p* < 0.05) (Table 6).

**Table 6:** Risk of postoperative complications with dislocation

Complication	OR	95% CI	*p*
Stroke/CVA	2.894	(1.181, 7.093)	**0.020**
Bleeding Transfusions	5.092	(4.475, 5.794)	** < 0.001**
Sepsis	3.232	(1.887, 5.536)	** < 0.001**

## Discussion

Early identification and recognition of preoperative risk factors for THA dislocations is an important component of interdisciplinary surgical planning and physician–patient communication on expected outcomes [3, 20, 21]. With the recent emphasis on value-based healthcare models, it is essential for surgeons to adequately optimize patients prior to surgery to reduce inpatient costs, facilitate speedy functional rehabilitation, reduce hospital readmissions, and decrease LOS [22]. While the surgical techniques and implants have advanced to decrease dislocation rates in susceptible elderly patients, there is still a component of pre-operative lab values, medical history, and perioperative care that needs to be further explored and standardized. Elderly patients undergoing arthroplasty are susceptible to dislocation complications due to relative medical frailty and are prone to underlying disabilities and fatigue limiting their quality of life and ability to return to functional independence [23]. This study found American Indian ethnicity, hypoalbuminemia, moderate to severe anemia, general anesthesia, increased operative time, and longer inpatient hospital stay to be independent risk factors for postoperative dislocation within 30 days. Risk stratification and medical clearance are essential components of surgical planning and optimization surgeons can take to prevent acute dislocations and reduce overall healthcare costs.

When baseline demographics were compared against non-dislocated patients, patients with 30-day postoperative hip dislocations were more likely to be females, older age, higher ASA classification, lower BMI, diabetics, smokers, and have a history of hypertension, bleeding disorders, and COPD. Similar to prior studies, older age, higher ASA, and COPD predict overall frailty among these patients which may contribute to underlying poor muscular resistance and tension needed to prevent dislocation events [24]. Smokers, diabetics, and patients with COPD have previously been shown to have increased prosthesis-related complications, including aseptic loosening, infections, and revisions which may stem from poor circulatory function and bone-implant integration leading to dislocation [25]. While some studies found a higher BMI to be a risk factor for dislocation possibly due to extra-articular impingement from thigh-on-thigh soft tissue contact during head adduction and flexion, our study suggested a lower BMI may instead be more susceptible to future dislocations [26]. A low BMI, especially < 18.5 is more likely to predict muscle weakness leading to ligamentous laxity, while obesity is associated with limited mobility and hence less risk of dislocations due to generalized immobility and disuse [27].

On multivariate analysis, American Indian ethnicity was an independent risk factor for sustaining a postoperative hip dislocation. Although prior studies have suggested White ethnicity to increase the risk of dislocation, there are limited studies on ethnic disparities, and disparities in THA are still relatively unknown with a lack of robust data. While a prior study has shown minorities to have an increased LOS and American Indians specifically with increased THA reoperation rates, our study suggests the inherent risk of dislocation in American Indians may be the cause for the higher return to the operating room [28]. American Indian patients have some of the highest adverse health outcomes after total knee arthroplasty, and future studies are needed to address ethnic disparities in THA causing postoperative dislocations that may be prevented by understanding underlying social determinants of health [29]. Socioeconomic status, access to healthcare, cultural beliefs, and neighborhood are currently discussed topics that are starting to become integrated into the perioperative optimization and comprehensive value-based approach to healthcare [30].

Hypoalbuminemia has been regularly used in the orthopedic literature to suggest malnutrition and frailty. While severely low GFR, history of renal complications, and recent weight loss > 10% predicted high rates of dislocation, only hypoalbuminemia was an independent risk factor for dislocation on multivariate linear regression. Hypoalbuminemia is considered to represent inadequate nutritional status and chronic inflammation leading to poor muscle mass and strength [31]. The decreased strength and muscle weakness likely contribute to increased dislocation risk due to imbalance, inability to comply with activity restrictions, and poor lower chain mobility with dynamic sit to stand maneuvers [32]. Our patients with hypoalbuminemia and malnutrition likely required increased hospitalization stay and discharge to acute rehabilitation for dependent gait assistance, strengthening, and safety precaution. In fact, increased LOS and a non-home discharge destination were also independent risk factors for sustaining a postoperative dislocation on multivariate analysis. A decreased LOS and discharge home not only help reduce health care utilization costs but also improve recovery in the comfort of the patient’s home by decreasing iatrogenic complications and infections. Although our study investigated the utility of a 5-Factor Modified Frailty Index on predicting dislocation risk, there was no significant correlation between the mFI-5 and prediction of 30-day dislocation [19]. Implementation of a postoperative protein-based diet after hip fracture surgery has previously been associated with lower complication rates, and surgeons should consider nutrition consultation, vitamin supplementation, and emphasis on a high-protein diet to decrease dislocation rate, decrease frailty, improve muscular strength, and reduce overall medical complications [33].

Preoperative lab markers are routinely ordered to stratify at risk patients for medical complications, but there are few studies examining risk factors and values that predict propensity for dislocations [34]. In this study, a low eGFR, hyponatremia, BUN/Cr > 25, high alkaline phosphatase levels, leukocytosis, anemia, and a high INR level were associated with postoperative dislocation events (Table 4). However, it is important to note that the INR was statistically significant, a difference of 0.1 is likely not clinically significant. GFR and hyponatremia are preoperative risk factors that reflect underlying fluid balance and circulatory deficiencies that increase the risk of postoperative falls, delirium, cognitive impairment, and medical complications that increase the risk for dislocation [35]. A high BUN/Cr > 25 is a sensitive marker for dehydration, and dehydrated patients in the post-surgical period are prone to underlying disabilities and fatigue, which may preclude safe, proper adherence to postoperative posterior hip precautions for preventing dislocation [36]. In addition, high alkaline phosphatase levels may reflect an underlying abnormality in bone quality and density that may not be able to withstand stresses from prosthetic impaction, reaming, broaching, and early weight bearing [37]. Perhaps due to poor bone healing and underlying malnourishment, our high alkaline phosphatase, hyponatremic, dehydrated, and coagulopathic patients had an increased risk of postoperative dislocations from overall frailty and poor soft tissue integrity. In fact, among our preoperative lab values, multivariate analysis revealed anemia to be an independent risk factor for postoperative dislocations. Anemic patients are susceptible to post anesthetic and stresses of surgery, and they are prone to sustaining falls and orthostatic hypotensive episodes leading to dislocation [38]. Proper preoperative treatment of anemia may reduce postoperative weakness and fatigue that may lead to improved balance, rehabilitation, and gait training needed to decrease dislocation rates.

In addition to the preoperative risk stratification of patients who may be susceptible to complications, it is important for surgeons to identify perioperative factors, such as type of anesthesia and operative time, as independent risk factors for dislocation. In our multivariate logistic regression, general anesthesia and increased operative time were significantly associated with dislocation events. General compared to spinal anesthesia for hip surgery has previously been shown to decrease mortality, thromboembolic events, blood loss, pulmonary complications, and transfusion requirements [39]. Decreased postoperative complications may allow for early functional rehabilitation and decreased length of inpatient stay, further decreasing the rate of dislocation events. A prior study has examined the possible benefits of a motor blockade and resultant soft tissue laxity seen intraoperatively during THA on spinal anesthesia patients that lead to perceived vertical offset and further soft tissue tensioning leading to overall decreased dislocation rates [40]. Furthermore, increased operative time was also an independent risk factor for dislocation in this study, possibly due to overall increased anesthesia combined with difficulty of the case leading to higher dislocation rates [41].

Not only are readmissions and reoperation rates for THA dislocations costly and increase morbidity, but patients presenting with dislocated THA are also at increased risk for sustaining further cerebrovascular accidents, bleeding requiring transfusion requirements, and sepsis. Periprosthetic dislocations cause increased tension on neurovascular structures and soft tissue disruption, leading to hemorrhage and deep hematoma formation [41]. The deep hematoma and underlying bleeding may cause acute blood loss anemia requiring postoperative transfusions, which are known to increase overall morbidity, outcomes, and infections in THA [42]. Stasis of the hematoma in combination with allogenic transfusions may lead to infections of the hip and further sepsis if not addressed immediately and carefully monitored. Perioperative immobilization and venous disruption caused by traumatic dislocation events may possibly also lead to higher risk of undiagnosed thromboembolism causing increased cerebrovascular accident rates as seen in this study.

Despite the large number of patients included, there are limitations to consider when using the NSQIP database, including selection bias. Although we were able to analyze all primary THA using CPT codes, there was unfortunately no ability to assess anterior versus posterior approach, conventional versus navigation assisted techniques, and the type of implants, such as dual mobility cups, constrained liners, or high offset stems. The anterior approach has gained increasing popularity for high-risk patients, and this national database was unable to differentiate concurrent spine pathology, which has been shown to be an independent risk factor for dislocation [43, 44]. While the data consists of a heterogeneous population nationwide at different ambulatory settings, the wide variety of in-patient hospitals and surgeon expertise may confound outcomes. Although various institutions may implement different preoperative pathways for joint arthroplasty, patients from both academic and private practice settings in rural and urban centers reflect the generalizability of our results. Furthermore, it is possible that we were not able to record all cases of postoperative periprosthetic dislocations as the database is limited to short 30-day complication rates. Lastly, the database does not include variables involving mental status or mental health, which is increasing important in modern healthcare.

Risk stratification and medical clearance are essential components of surgical planning to reduce dislocation events and decrease healthcare costs. Patients with preoperative hypoalbuminemia, moderate to severe anemia, American Indian ethnicity, and non-home discharge are at risk for sustaining acute dislocations after index THA. Perioperative risk factors, such as general anesthesia, increased operative time, and increased length of inpatient hospital stay are modifiable factors that further increase risk for dislocation in susceptible patients. Careful interdisciplinary planning and medical optimization should be considered in high-risk frail patients as postoperative dislocations significantly increase the risk of sepsis, CVA, and blood transfusions on readmission.

## Data Availability

The datasets generated and/or analyzed during the current study are available in the ACS National Surgical Quality Improvement Program (NSQIP) database at https://www.facs.org/quality-programs/acs-nsqip.

## Abbreviations

THA: Total hip arthroplasty
NSQIP: National Surgical Quality Improvement Program
CPT: Current Procedural Terminology
COPD: Chronic obstructive pulmonary disease
Cr: Creatinine
CKD: Chronic kidney disease
US: United States
BMI: Body mass index
eGFR: Estimated glomerular filtration rate
BUN: Blood urea nitrogen
INR: International normalized ratio
WBC: White blood cell
CHF: Congestive heart failure
PRBCs: Packed red blood cells
SIRS: Systemic inflammatory response syndrome
SSI: Surgical site infection
CVA: Cerebral vascular accident
DVT: Deep vein thrombosis
LOS: Length of stay
